# COVID-19 Vaccine Hesitancy Among Patients Recovered From COVID-19 Infection in Wuhan, China: Cross-Sectional Questionnaire Study

**DOI:** 10.2196/42958

**Published:** 2023-07-03

**Authors:** Yiman Huang, Ling Zhang, Jiaqi Fu, Yijin Wu, Hao Wang, Weijun Xiao, You Xin, Zhenwei Dai, Mingyu Si, Xu Chen, Mengmeng Jia, Zhiwei Leng, Dan Cui, Xiaoyou Su

**Affiliations:** 1 School of Population Medicine and Public Health Chinese Academy of Medical Sciences & Peking Union Medical College Beijing China; 2 National Clinical Research Center for Respiratory Diseases China-Japan Friendship Hospital Beijing China

**Keywords:** COVID-19, COVID-19 survivors, vaccine hesitancy, complacency, confidence, convenience, cross-sectional questionnaire, health education, health promotion, public health

## Abstract

**Background:**

Although patients recovered from COVID-19 already have immunity gained from natural infection, they are still at risk of reinfection due to the emergence of new variants of COVID-19 and the diminishing of naturally acquired immunity over time. Vaccination is associated with efficacious protection against COVID-19 infection and could boost infection-acquired immunity; however, various COVID-19 survivors have not been vaccinated due to vaccine hesitancy.

**Objective:**

The aim of this study was to investigate COVID-19 vaccine hesitancy and related factors among COVID-19 survivors.

**Methods:**

A cross-sectional questionnaire survey was conducted among patients who recovered from COVID-19 infection in Wuhan, China, between June 10 and July 25, 2021. The questionnaire included sociodemographic information, items on COVID-19 infection, the COVID-19 vaccine hesitancy scale based on the 3Cs (complacency, convenience, and confidence) model, trust in vaccine manufacturers and health facilities, and reasons for the decision to accept COVID-19 vaccination. Multivariate logistic regression analysis was used to assess the factors influencing COVID-19 vaccine hesitancy.

**Results:**

Among the 1422 participants, 538 (37.8%) were not vaccinated against COVID-19. The COVID-19–recovered patients who self-reported having a current unhealthy status expressed more hesitancy about the COVID-19 vaccine than those who perceived themselves to be healthy (odds ratio [OR] 0.45, 95% CI 0.28-0.71). Compared to the asymptomatic patients, patients with mild symptoms were more likely to receive a COVID-19 vaccine (OR 1.67, 95% CI 1.02-2.82). Regarding the 3Cs model, high complacency (*P=*.005) and low convenience (*P=*.004) were significant negative factors for COVID-19 vaccination. Trust in vaccine manufacturers and health facilities was a significant positive factor for COVID-19 vaccination (OR 1.14, 95% CI 1.09-1.19). “Self-needs” was the main reason for patients to receive the COVID-19 vaccine, whereas “already have antibodies and do not need vaccination” was the main reason for patients to not receive the COVID-19 vaccine.

**Conclusions:**

Among the three major factors of vaccine hesitancy, complacency proved to be the most notable among COVID-19–recovered patients. Therefore, educational campaigns can focus on raising the awareness of risk of infection and the benefits of vaccination to reduce complacency toward vaccination among this population. In particular, for individuals who have recovered from COVID-19, improving factors related to convenience such as transportation, the environment of vaccination, and providing door-to-door service was also deemed necessary to facilitate their vaccination. In addition, addressing the concerns about vaccination of COVID-19–recovered patients could foster trust and promote their uptake of vaccination.

## Introduction

The COVID-19 pandemic has caused a serious disease burden, as well as financial, psychological, and life hardship for people over the past 3 years. Currently, the high transmissibility, high pathogenicity, and high immune-evasion ability of the emerging new variants pose new challenges and uncertainties in preventing COVID-19 infection, as well as posing a serious threat to public health [1-3]. Previous studies have demonstrated that individuals naturally infected with COVID-19 might be at decreased future risk of COVID-19 infection due to the immunity induced by contracting COVID-19 to protect them from reinfection [4]. However, according to the World Health Organization (WHO), the presence of antibodies in recovered patients does not guarantee protection against reinfection, and the probability of protection against reinfection at 6 months was estimated to be 50.0% in people aged over 65 years [5-7]. Reinfection of COVID-19 could be as severe or even more severe than the first infection, including among patients with antibodies [6,8]. Therefore, since previous infection does not necessarily protect an individual from reinfection, it is necessary for patients who recovered from COVID-19 infection to protect against reinfection through COVID-19 vaccination [9].

Vaccination is considered to be one of the greatest achievements of public health. The ongoing COVID-19 pandemic can be mitigated by an efficacious vaccine, which reduces disease incidence, prevalence, new hospitalizations, and intensive care demand [10,11]. The COVID-19 vaccine has been shown to provide significant protection against COVID-19 infection and to significantly reduce the risk of symptomatic COVID-19 infection. Moreover, COVID-19 vaccination could boost infection-acquired immunity, and this increased immunity would remain high for more than 1 year after infection [12,13]. In 2021, five COVID-19 vaccines were approved for conditional marketing or emergency use in China, including inactivated vaccines, adenovirus vector vaccines, and recombinant protein vaccines, and the Chinese government provided nationwide COVID-19 vaccination for all populations free of charge. As of July 25, 2021, approximately 155 million cumulative doses of the COVID-19 vaccine had been administered in mainland China [14]. With the steady increase in COVID-19 vaccine supplies, vaccine hesitancy is becoming a barrier to high vaccine coverage [15]. There are still many people who delay or reject being vaccinated due to vaccine hesitancy even though they are recommended to accept the COVID-19 vaccination, including COVID-19 survivors. In the United States, vaccination coverage (>1 dose and full vaccination) was lower among those who ever had COVID-19 than among those who had no history of COVID-19 infection from July to August 2021 [16]. In Italy, 34.2% and 24.9% of COVID-19 patients were undecided or reluctant, respectively, to receive the COVID-19 vaccine from September to November 2020 [17].

The SAGE Working Group on Vaccine Hesitancy defines vaccine hesitancy as a delay in acceptance or refusal of vaccination despite the availability of vaccination services. Vaccine hesitancy is complex and context-specific, varying across time, place, and vaccines [18]. Factors such as the evolving epidemiological context and multiple waves of infection, trust in the health care system, attitudes toward vaccines, self-efficacy, and the presence of chronic disease have all been indicated to be associated with hesitancy to receive the COVID-19 vaccine [19-23]. In particular, COVID-19–recovered patients might experience not only physical disease symptoms but also psychological distress [24,25]. Some of these individuals might be hesitant to get vaccinated, fearing that they are not physically able to tolerate the vaccine. In addition, since other infectious diseases such as measles confer immunity for a longer duration or for life, some patients might consider that infection-acquired immunity with COVID-19 is the best type of immunity. Moreover, the concerns about the safety of vaccines developed in a short time might also be a factor contributing to COVID-19 vaccine hesitancy [26].

Vaccine hesitancy as a complex decision-making process has been interpreted based on various conceptual models such as the “3Cs” model, the health belief model, and the theory of planned behavior [27,28]. The “3Cs” model is one of the most widely known models, highlighting three dimensions: complacency, referring to the belief that perceived risks of vaccine-preventable diseases are low and that vaccination is not a necessary preventive action; convenience, referring to vaccine availability and accessibility; and confidence, referring to the trust in the effectiveness and safety of vaccines, the delivery system, and the motivations of vaccination policy makers [29]. Previous studies demonstrated that complacency, convenience, and confidence were equally significant factors influencing COVID-19 vaccine hesitancy [30-32].

Given the lack of data on COVID-19 vaccination status among COVID-19–recovered patients infected with the original SARS-CoV-2 strain in China, the aim of this study was to investigate COVID-19 vaccine hesitancy status and relevant factors among COVID-19–recovered patients. In particular, we focused on the role of the factors considered in the 3Cs model to identify the specific constructs that can influence COVID-19 vaccine hesitancy. In addition, the vaccination status and possible determinants of vaccine hesitancy in terms of sociodemographic information; clinical classification; history of infection in family members, relatives, and friends; and other vaccination history were investigated among COVID-19–recovered patients. This study will provide a basis for developing vaccination promotion activities and strategies for COVID-19–recovered patients to reduce their vaccine hesitancy and promote them to receive the COVID-19 vaccine.

## Methods

### Study Design and Sampling

This cross-sectional study was carried out among former COVID-19 patients in Jianghan District of Wuhan, China, from June 10 to July 25, 2021. According to the electronic medical records of the Health Bureau of Jianghan District and the inclusion criteria, a total of 3059 recovered COVID-19 patients were eligible for the study who had all been infected with the original SARS-CoV-2 strain and were diagnosed between December 10, 2019, and April 20, 2020. Among them, 1601 COVID-19 survivors were invited to complete a questionnaire survey on their vaccine hesitancy status when they were receiving clinical reexamination. If they agreed to participate in this survey, they were invited to the “Survey Room” to complete the questionnaire. Self-administered electronic questionnaires were generated on Research Electronic Data Capture (REDCap), an online survey platform, from which patients could complete by themselves. To ensure the quality of the survey, our trained investigators stayed at the “Survey Room” to promptly answer the participants’ questions.

The following inclusion requirements had to be met by participants: participants must be at least 18 years old, have a COVID-19 infection history, be able to work independently with the researcher to complete several scale assessments, and have access to a mobile communication device such as a cell phone with a WeChat account. People who fit one or more of the following criteria were excluded: (1) having significant cognitive impairment; (2) having life-threatening medical conditions such as heart, lung, kidney, liver diseases or cancers; and (3) finding it challenging to cooperate with the questionnaire study. In total, 1422 of the 1601 invited participants were chosen as the sample for this study based on the aforementioned standards and after discarding any incomplete questionnaires.

### Ethical Considerations

Ethics approval for the study was obtained from the Ethics Review Committee of the Institute of Pathogen Biology, Chinese Academy of Medical Sciences, Beijing, China (IPB-2020-22). All individuals provided digital informed consent to ensure their voluntary participation, which also included consent that the study data could be analyzed when used anonymously. All data were deidentified. All data are stored in an account with a password and cannot be used without consent. All study participants were compensated for transportation to the questionnaire site.

### Measurements

#### Sociodemographic Characteristics

Demographic characteristics included gender, age, place of residence, education level, marital status, smoking (whether or not they are habitual smokers), drinking (whether or not they habitually drink alcohol), and perceived current health status. We also investigated COVID-19 clinical classification by asking participants which type of diagnosis they had after their first admission (ie, asymptomatic, mild, moderate, clinically severe) and their acute-phase symptoms (ie, fever, respiratory symptoms, cardiovascular symptoms, gastrointestinal symptoms, other). Questions on COVID-19 infection and vaccination were also surveyed, including history of infection in family members, history of infection in relatives and friends, and other vaccination history (within the last 5 years).

#### COVID-19 Vaccine Hesitancy Scale Based on the 3Cs Model

On the basis of the flu vaccine hesitancy scale, we replaced “flu vaccine” in the items with “COVID-19 vaccine” to form the revised COVID-19 vaccine hesitancy scale. The flu vaccine hesitancy scale consists of 6 items and the 3 dimensions of the 3Cs model (complacency, confidence, and convenience) [33]. Complacency was measured by perceived necessity and importance of the vaccine, confidence was measured by perceived vaccine safety and effectiveness, and convenience was measured by perceived convenience and affordability of the vaccine. Participants rated each item on a 5-point Likert scale (1=strongly disagree; 5=strongly agree). The items were as follows: (1) necessity (“Thinking specifically about the COVID-19 vaccine, do you think the COVID-19 vaccine is necessary?”), (2) importance (“Thinking specifically about the COVID-19 vaccine, do you think the COVID-19 vaccine is important?”), (3) safety (“Thinking specifically about the COVID-19 vaccine, do you think the COVID-19 vaccine is safe?”), (4) effectiveness (“Thinking specifically about the COVID-19 vaccine, do you think the COVID-19 vaccine is effective?”), (5) convenience (“Thinking specifically about the COVID-19 vaccine, do you think the COVID-19 vaccine is convenient?”), and (6) affordability (“Thinking specifically about the COVID-19 vaccine, do you think the COVID-19 vaccine is affordable?”). The Cronbach α value for the whole scale was .92, and the Cronbach α values for the dimensions of complacency, convenience, and confidence were .92, .94, and .73, respectively. [34].

#### Trust

Two items were used to measure participants’ trust in vaccine manufacturers and health facilities, respectively, which were each scored on a 10-point Likert scale (1=strongly disagree; 10=strongly agree). The Cronbach α value for the two items was .92.

#### Reasons for Accepting and Not Accepting the COVID-19 Vaccine

For participants who had received the COVID-19 vaccine, multiple-choice questions were designed to investigate the corresponding reasons, with answers including: (1) self-needs, (2) recommended by health agencies, (3) recommended by others (eg, relatives, friends, neighbors), (4) recommended by those who have been vaccinated, (5) internet information, (6) free vaccination, and (7) others.

For participants who had not received the COVID-19 vaccine, multiple-choice questions were set up to investigate the reasons, with answers including: (1) don’t know how to get reliable information about COVID-19 vaccine, (2) have received negative media reports about COVID-19 vaccines, (3) have had a bad experience with the health clinic or provider, (4) have had a bad experience or adverse reactions from previous vaccinations, (5) have been told that others have had adverse reactions from some vaccines, (6) fear of injections, (7) already have antibodies and do not require vaccination, (8) long waiting time for vaccination, and (9) others.

### Statistical Analysis

#### Confirmatory Factor Analysis of the COVID-19 Vaccine Hesitancy Scale

To validate the COVID-19 vaccine hesitancy scale, we performed a confirmatory factor analysis. Model fit was evaluated by various goodness-of-fit indices, including the root mean square error of approximation (RMSEA), comparative fit index (CFI), and Tucker Lewis index (TLI) [35-37]. RMSEA values close to 0.06 or below were regarded as a good fit, 0.07 to 0.08 as moderate fit, 0.08 to 0.10 as marginal fit, and >0.10 as poor fit [38]. For the CFI and TLI, values close to 0.95 or above were regarded as good fit, values close to 0.90 and 0.95 as acceptable fit, and values approaching 0 as poor fit [39,40]. Convergent validity was assessed by average variance extracted (AVE) and composite reliability (CR), and convergent validity was considered high if the AVE was greater than 0.50, CR was greater than 0.70, and CR was greater than AVE. Discriminant validity was considered satisfactory if the correlation between the factor scores was significant and the correlation coefficient was less than the square root of the corresponding AVE [41,42].

#### Univariate and Multivariate Analysis

Categorical variables are summarized as frequencies and proportions, whereas continuous variables are summarized as mean (SD). Respondents who were vaccinated against COVID-19 indicated that they were not hesitant about the COVID-19 vaccine, whereas those who were not vaccinated against COVID-19 indicated that they had COVID-19 vaccine hesitancy. To identify any differences in the distributions of variables between the vaccinated and unvaccinated groups, the *χ*^2^ test and *t* test were used for categorical and continuous variables, respectively. Using the unvaccinated population as a reference, multiple logistic regression analysis was used to assess the association between the investigated factors and COVID-19 vaccine hesitancy. The associations between dependent and independent variables were determined using the odds ratio (OR) with 95% CI and a *P* value <.05 was deemed to indicate statistical significance of the independent variables. Data were analyzed using Stata version 16.0.

## Results

### Confirmatory Factor Analysis of the COVID-19 Vaccine Hesitancy Scale

**Figure 1:**
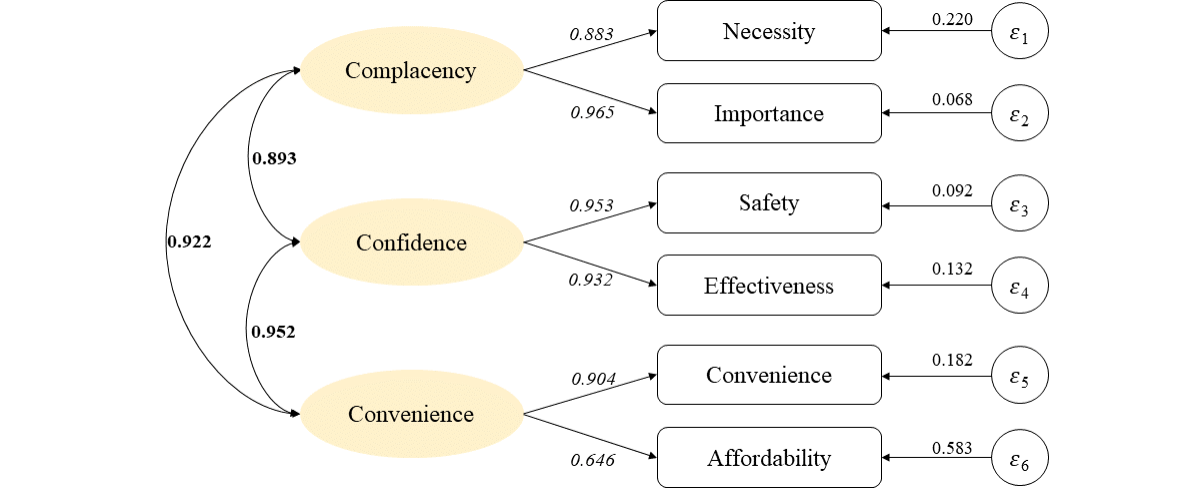
Results of the confirmatory factor analysis model. Standardized covariances between dimensions are shown in bold, standardized path coefficients are italicized, and standardized variances are also shown.

Figure 1 depicts the results of the confirmatory factor analysis for the model built in the sample, including the three dimensions of the 3Cs model. Separate confirmatory factor analysis based on the original structure of the “complacency” dimension (2 items), “confidence” dimension (2 items), and “convenience” dimension (2 items) were performed. The RMSEA value (0.076) indicated a moderate fit, the CFI value (0.994) indicated a good fit, and the TLI value (0.986) indicated a good fit.

Table 1 shows the results of convergent validity and discriminant validity of the model. For convergent validity, the AVE values for all dimensions of the model were greater than 0.50 and the CR values for all three dimensions of the model were greater than 0.70. In addition, the CR values for all factors in the model were greater than the AVE. For discriminant validity, the correlation coefficients for any two dimensions were less than the square root of the corresponding AVE, except for the correlation coefficients for confidence and convenience, which were higher than the square root of the corresponding AVE.

**Table 1:** Convergent and discriminant validity of the COVID-19 vaccine hesitancy model.

Dimensions	Pearson correlation coefficient			AVE^a^		CR^b^
	Complacency	Confidence	Convenience			
Complacency	*0.925^c^*	N/A^d^	N/A	0.855	0.922	
Confidence	–0.827^*^	*0.942*	N/A	0.888	0.941	
Convenience	–0.759^*^	0.796^*^	0.785	0.617	0.758	

^a^AVE: average variance extracted.

^b^CR: composite reliability.

^c^Values on the diagonal are the square roots of each AVE value for comparison with other correlation coefficients; values in italics indicate a higher square root of the AVE value in each subscale than the correlation coefficients with other subscales.

^d^N/A: not applicable.

^*^P<.001.

### Sociodemographic Characteristics of Participants

Among the 1422 participants, 538 (37.8%) were not vaccinated against COVID-19. There was a higher proportion of participants aged 41 to 60 years (634/1422, 45.2%), followed by the 61-80 years age group (611/1422, 43%). The great majority of the participants lived in urban areas (1245/1422, 87.6%), and 1197 (84.2%) participants were married. The majority (968/1422, 68.1%) had an education level of senior high school or below. Nearly half of the participants had a household income for 2020 lower than 60,000 Yuan (approximately US $8607) per year. Most participants self-reported that they were not current smokers (1233/1422, 86.7%) or drinkers (1038/1422, 73.0%), and 6.1% (87/1422) of the patients were asymptomatic, 70.8% (1007/1422) had mild symptoms of COVID-19, 10.3% (146/1422) had moderate symptoms, and 12.8% (182/1422) had critically severe symptoms. In the acute phase, 62.7% (891/1422) of patients had fever, 49.9% (709/1422) had respiratory symptoms, 18.2% (259/1422) had gastrointestinal symptoms, 6.4% (91/1422) had cardiovascular symptoms, and 15.5% (221/1422) had other symptoms. Overall, 24.1% (342/1422) were reinfected with COVID-19 after the first discharge, nearly half of the participants (654/1422, 46%) had family members who had been infected with COVID-19, 30.2% (429/1422) had relatives or friends who had been infected with COVID-19, and 17.2% (245/1422) of the participants had received other vaccines within the last 5 years (Table 2).

Table 2 also shows that the age gap between vaccinated and unvaccinated participants was statistically significant; participants aged ≤40 years had a higher hesitancy rate compared to the other three age groups (*P*<.001). The hesitancy rate of those living in urban areas (485/1245, 39.0%) was significantly higher than that of participants living in a village (53/177, 29.9%). Significantly, participants who were married had a lower hesitancy rate (428/1197, 35.8%) compared to that of participants who were single (23/54, 42.6%) and those with another marital status (87/171, 50.9%). Participants who perceived having an unhealthy status had a higher hesitancy rate than that of participants who self-reported being healthy (*P*<.001). Patients who had fever in the acute phase had a higher COVID-19 vaccine hesitancy rate (359/891, 40.3%) than those who did not have fever (179/531, 33.7%).

**Table 2:** Sociodemographic characteristics of participants.

Variables				Unvaccinated (n=538)		Vaccinated (n=884)		Total (N=1422)		*P* value	
**Gender, n (%)**											.45
	Male			221 (36.7)		381 (63.3)		602 (42.3)			
	Female			317 (38.7)		503 (61.3)		820 (57.7)			
**Age (years), n (%)**											<.001
	≤40			70 (48.3)		75 (51.7)		145 (10.2)			
	41-60			240 (37.3)		403 (62.7)		643 (45.2)			
	61-80			207 (33.9)		404 (66.1)		611 (43.0)			
	≥81			21 (87.5)		2 (12.5)		23 (1.6)			
**Residence place, n (%)**											.02
	Urban			485 (39.0)		760 (61.0)		1245 (87.6)			
	Village			53 (29.9)		124 (70.1)		177 (12.4)			
**Marital status, n (%)**											.001
	Single			23 (42.6)		31 (57.4)		54 (3.8)			
	Married			428 (35.8)		769 (64.2)		1197 (84.2)			
	Others			87 (50.9)		84 (49.1)		171 (12.0)			
**Education level, n (%)**											.02
	Senior high school or below			347 (35.9)		621 (64.1)		968 (68.1)			
	College and above			191 (42.1)		263 (57.9)		454 (31.9)			
**Income for 2020 (CNY^a^/year), n (%)**											.20
	<60,000 Yuan			310 (36.9)		531 (63.1)		841 (59.1)			
	60,000-120,000 Yuan			135 (36.5)		235 (63.5)		370 (26.0)			
	130,000-300,000 Yuan			84 (44.9)		103 (55.1)		187 (13.2)			
	>300,000 Yuan			9 (37.5)		15 (62.5)		24 (1.7)			
**Have underlying diseases, n (%)**											.06
	Yes			315 (40.0)		473 (60.0)		788 (55.4)			
	No			223 (35.2)		411 (64.8)		634 (44.6)			
**Perceived current health status, n (%)**											<.001
	Healthy			482 (36.5)		840 (63.5)		1322 (93.0)			
	Unhealthy			56 (56.0)		44 (44.0)		100 (7.0)			
**Current smoker, n (%)**											.38
	Yes			77 (40.7)		112 (59.3)		189 (13.3)			
	No			461 (37.4)		772 (62.6)		1233 (86.7)			
**Alcohol use, n (%)**											.31
	Yes			137 (35.7)		247 (64.3)		384 (27.0)			
	No			401 (38.6)		634 (61.4)		1038 (73.0)			
**Clinical classification of COVID-19 patients, n (%)**											.17
	Asymptomatic			39 (44.8)		48 (55.2)		87 (6.1)			
	Mild			363 (36.1)		644 (63.9)		1007 (70.8)			
	Moderate			60 (41.1)		86 (58.9)		146 (10.3)			
	Critically severe			76 (41.8)		106 (58.2)		182 (12.8)			
**Acute-phase symptoms, n (%)**											
	**Fever**										.01
		Yes	359 (40.3)		532 (59.7)		891 (62.7)				
		No	179 (33.7)		352 (66.3)		531 (37.3)				
	**Respiratory symptoms**										.85
		Yes	270 (38.1)		439 (61.9)		709 (49.9)				
		No	268 (37.6)		445 (62.4)		713 (50.1)				
	**Gastrointestinal symptoms**										.48
		Yes	103 (39.8)		156 (60.2)		259 (18.2)				
		No	435 (37.4)		728 (62.6)		1163 (81.8)				
	**Cardiovascular symptoms**										.59
		Yes	32 (35.2)		59 (64.8)		91 (6.4)				
		No	506 (38.0)		825 (62.0)		1331 (93.6)				
	**Other symptoms**										.59
		Yes	80 (36.2)		141 (63.8)		221 (15.5)				
		No	458 (38.1)		743 (61.9)		1201 (84.5)				
**Reinfection of COVID-19 after the first discharge, n (%)**											.23
	Yes			139 (40.6)		203 (59.4)		342 (24.0)			
	No			323 (37.9)		530 (62.1)		853 (60.0)			
	Not hospitalized (not sure)			76 (33.5)		151 (66.5)		227 (16.0)			
**Have family members infected with COVID-19, n (%)**											.48
	Yes			241 (36.9)		413 (63.1)		654 (46.0)			
	No			297 (38.7)		471 (61.3)		768 (54.0)			
**Have relatives or friends infected with COVID-19, n (%)**											.22
	Yes			152 (35.4)		277 (64.6)		429 (30.2)			
	No			386 (38.9)		607 (61.1)		993 (69.8)			
**Have received other vaccinations within the last 5 years, n (%)**											.07
	Yes			80 (32.7)		165 (67.3)		245 (17.2)			
	No			458 (38.9)		719 (61.1)		1177 (82.8)			

^a^CNY: Chinese Yuan renminbi (1 CNY was equivalent to approximately US $6.98 in 2020).

### Contributions of 3Cs Model Constructs and Trust to Vaccine Hesitancy

**Figure 2:**
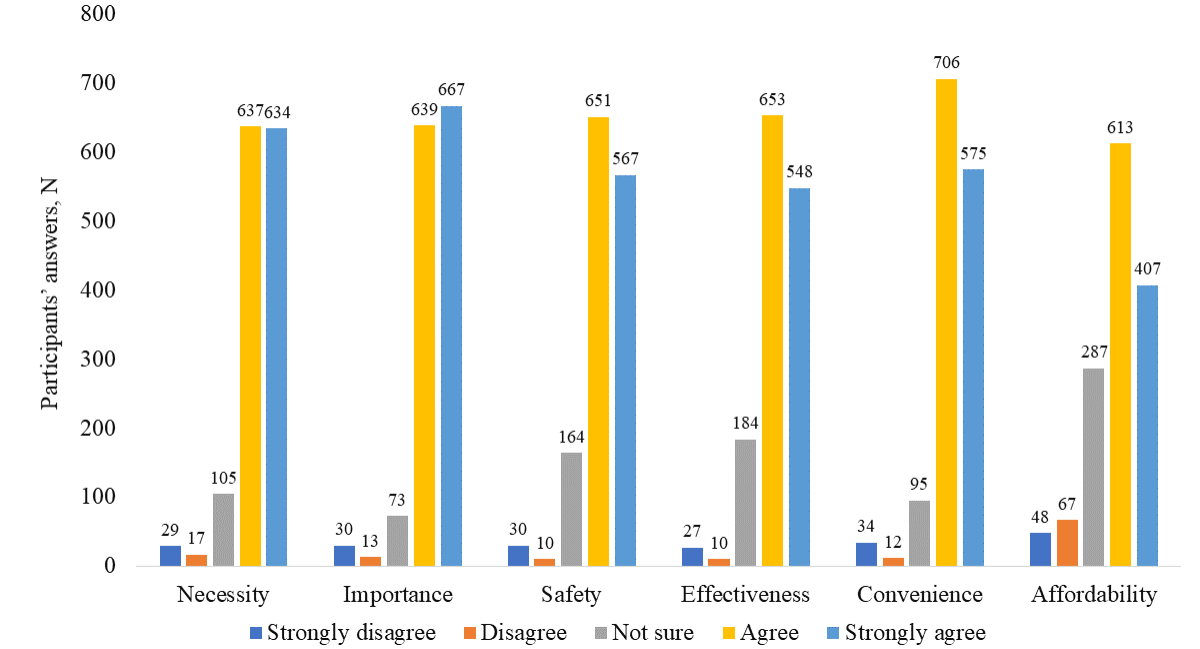
Distribution of answers on the COVID-19 vaccine hesitancy scale according to the 3Cs model (complacency, convenience, confidence).

Figure 2 shows the distribution of answers on the COVID-19 vaccine hesitancy scale based on the 3Cs model among all participants. Table 3 shows the univariate analysis results of 3Cs model components and trust in vaccinated and unvaccinated groups. The unvaccinated group scored significantly higher on “complacency” related to COVID-19 vaccination than the vaccinated group. In contrast, participants who had received the COVID-19 vaccine scored higher on “confidence” and “convenience” than those who were not vaccinated. Moreover, the vaccinated group scored significantly higher than the unvaccinated group on trust (all *P*<.001).

**Table 3:** Univariate analysis of the 3Cs (complacency, confidence, convenience) model constructs and trust in vaccinated and unvaccinated groups.

Variables	Scores, mean (SD)				*P* value
	Unvaccinated (n=538)	Vaccinated (n=884)	Total (N=1422)		
3Cs Complacency	3.82 (1.69)	3.11 (1.40)	3.38 (1.55)	<.001	
3Cs Confidence	7.96 (1.65)	8.66 (1.52)	8.39 (1.61)	<.001	
3Cs Convenience	7.66 (1.62)	8.43 (1.52)	8.14 (1.60)	<.001	
Trust	17.02 (3.39)	18.42 (2.44)	17.89 (2.91)	<.001	

### Multivariate Predictors of COVID-19 Vaccine Hesitancy

Table 4 shows that participants aged 41-60 years (OR 2.12, 95% CI 1.34-3.36) and 61-80 years (OR 2.59, 95 %CI 1.56-4.30) were more likely to receive a COVID-19 vaccine than participants aged ≤40 years; however, participants aged >80 years (OR 0.14, 95% CI 0.03-0.66) were less likely to receive a COVID-19 vaccine than those aged <40 years. Participants who reported “other” marital status (ie, divorced, widowed) were more likely to be hesitant about receiving the COVID-19 vaccine than those who were single (OR 0.42, 95% CI 0.19-0.91). In addition, patients who perceived having an unhealthy status had more hesitancy about the COVID-19 vaccine than those who were healthy (OR 0.45, 95% CI 0.28-0.71). Compared to the asymptomatic patients, patients with mild symptoms were more likely to receive the COVID-19 vaccine (OR 1.67, 95% CI 1.02-2.82). Regarding the acute-phase symptoms, patients who did not have fever were less likely to receive the COVID-19 vaccine than those who had fever (OR 1.48, 95% CI 1.13-1.94). Not receiving any other vaccination within the last 5 years was negatively associated with receiving the COVID-19 vaccine (OR 0.69, 95% CI 0.50-0.95). Regarding the 3Cs model constructs, complacency was a significant negative factor for getting vaccinated for COVID-19 (OR 0.81, 95% CI 0.71-0.94), whereas convenience was a significant positive factor for COVID-19 vaccination (OR 1.21, 95% CI 1.06-1.37). Participants who had more trust were more likely to receive the COVID-19 vaccine (OR 1.14, 95% CI 1.09-1.19).

**Table 4:** Multivariate logistic regression analysis on factors contributing to COVID-19 vaccine hesitancy.^a^

Variables		OR^b^ (SE)	95% CI	*P* value	
**Gender**					
	Male (reference)	N/A^c^	N/A	N/A	
	Female	0.85 (0.12)	0.63-1.13	.26	
**Age (years)**					
	≤40 (reference)	N/A	N/A	N/A	
	41-60	2.12 (0.50)	1.34-3.36	.001	
	61-80	2.59 (0.67)	1.56-4.30	<.001	
	≥81	0.14 (0.11)	0.03-0.66	.01	
**Place of residence**					
	Urban (reference)	N/A	N/A	N/A	
	Village	1.45 (0.29)	0.98-2.16	.07	
**Marital status**					
	Single (reference)	N/A	N/A	N/A	
	Married	0.73 (0.26)	0.37-1.45	.37	
	Others	0.42 (0.17)	0.19-0.91	.03	
**Education level**					
	Senior high school or below (reference)	N/A	N/A	N/A	
	College and above	0.85 (0.14)	0.63-1.18	.36	
**Income for 2020 (CNY^d^/year)**					
	<60,000 (reference)	N/A	N/A	N/A	
	60,000-120,000	1.00 (0.15)	0.75-1.34	>.99	
	130,000-300,000	0.77 (0.16)	0.52-1.15	.20	
	>300,000	0.88 (0.42)	0.35-2.22	.79	
No underlying disease		1.32 (0.17)	1.02-1.70	.03	
**Perceived current health status**					
	Healthy (reference)	N/A	N/A	N/A	
	Unhealthy	0.45 (0.11)	0.28-0.71	.001	
Nonsmoker		1.23 (0.24)	0.84-1.80	.28	
No alcohol use		0.82 (0.13)	0.61-1.11	.20	
**Clinical classification of COVID-19**					
	Asymptomatic	N/A	N/A	N/A	
	Mild	1.70 (0.44)	1.02-2.821	.04	
	Moderate	1.52 (0.49)	0.81-2.85	.19	
	Critically severe	1.42 (0.44)	0.77-2.62	.27	
**Acute-phase symptoms (yes=reference)**					
	No fever	1.48 (0.21)	1.13-1.94	.01	
	No respiratory symptoms	0.97 (0.12)	0.75-1.24	.79	
	No gastrointestinal symptoms	1.07 (0.17)	0.78-1.47	.67	
	No cardiovascular symptoms	0.69 (0.18)	0.41-1.15	.15	
	No other symptoms	0.89 (0.15)	0.41-1.15	.15	
	No reinfection of COVID-19 after first discharge	1.07 (0.16)	0.81-1.43	.64	
Not hospitalized (not sure)		1.50 (0.30)	1.01-2.22	.045	
No family members infected with COVID-19		0.92 (0.12)	0.72-1.18	.53	
No relatives or friends infected with COVID-19		0.83 (0.12)	0.63-1.10	.20	
No other vaccinations within 5 years		0.69 (0.11)	0.50-0.95	.02	
3Cs Complacency		0.81 (0.06)	0.71-0.94	.005	
3Cs Confidence		0.92 (0.07)	0.79-1.06	.26	
3Cs Convenience		1.21 (0.08)	1.06-1.37	.004	
Trust		1.14 (0.03)	1.09-1.19	<.001	

^a^Model fit indices: Pearson *χ*^2^=1417.44, *P*=.25; Hosmer-Lemeshow *χ*^2^=14.39, *P*=.07.

^b^OR: odds ratio.

^c^N/A: not applicable.

^d^CNY: Chinese Yuan renminbi (1 CNY was equivalent to approximately US $6.98 in 2020).

### Reasons for Accepting and Not Accepting COVID-19 Vaccination

Among the reasons for getting vaccinated against COVID-19, “self-needs” was chosen most frequently (n=625), followed by “recommended by health agencies” (n=191) and “free vaccination” (n=149). The reason for “recommended by those who have been vaccinated” was selected 37 times, which was only higher than the “others” response option (n=11). Among the reasons for not getting vaccinated against COVID-19, “antibodies are already in existence and do not require vaccination” was chosen most frequently (n=88), followed by “don’t know how to get reliable information about the COVID-19 vaccine” (n=53) and “others” (n=30). The reason “have had a bad experience with the health clinic or provider” was selected 4 times, which was the least frequently selected option (Table 5).

**Table 5:** Reasons for accepting and not accepting COVID-19 vaccination.

Reasons		Participants, n
**Accepting COVID-19 vaccination**		
	Self-needs	625
	Recommended by health agencies	191
	Recommended by others (eg, relatives, friends, neighbors)	73
	Recommended by those who have been vaccinated	37
	Internet information	40
	Free vaccination	149
	Others	11
**Not accepting COVID-19 vaccination**		
	Don’t know how to get reliable information about COVID-19 vaccine	53
	Have received negative media reports about COVID-19 vaccines	26
	Have a bad experience with the health clinic or provider	4
	Have a bad experience or adverse reactions from previous vaccinations	18
	Have been told that others have had adverse reactions from vaccines	29
	Fear of injections	25
	Antibodies are already in existence and do not require vaccination	88
	Long waiting time for vaccination	19
	Others	30

## Discussion

### Principal Results

The WHO indicated that antibodies in COVID-19–recovered patients do not guarantee protection against reinfection, making it necessary to receive the COVID-19 vaccine after natural infection [7]. However, diverse and effective preventive measures such as wearing a mask provided an alternative to prevent COVID-19 and might undermine the perceived need for vaccination to the point of developing an attitude of COVID-19 vaccine hesitancy [43,44]. This study revealed that the rate of COVID-19 vaccine hesitancy among patients who recovered from COVID-19 was 37.8%. According to previous research, this vaccine hesitancy rate is higher than that determined in a similar time period among Chinese patients with chronic diseases, including HIV infection (27.5%), cancer (24.1%), and inflammatory bowel disease (27.4%) [45-47]. Even though COVID-19–recovered patients and patients with chronic diseases are both vulnerable to COVID-19 reinfection based on their poor health conditions, COVID-19–recovered patients have a lower perceived risk of COVID-19 reinfection due to their belief that they already have antibodies gained from previous infection compared to patients with chronic diseases. Several studies also suggested lower vaccination intention among COVID-19–recovered individuals compared to uninfected individuals [48,49]. Research has shown that acquired immunity from COVID-19 infection would diminish over 6-8 months and protection against the new variant (Omicron variant) might be inadequate [50]; thus, COVID-19–recovered patients are still at risk of reinfection. The high perceived risk of contracting diseases is an essential determinant in overcoming vaccine hesitancy; that is, individuals who perceived a high risk of COVID-19 infection were assumed to adopt more preventive health behaviors to avoid or minimize health risks [51]. As the threat of reinfection increases due to continuously emerging new mutant strains, raising COVID-19–recovered patients’ awareness of the reinfection risk is essential to reduce their vaccine hesitancy.

In this study, COVID-19 patients with mild symptoms were more likely to receive the COVID-19 vaccine than asymptomatic patients. Similarly, previous studies have shown that people who have experienced severe COVID-19 disease have a lower rate of hesitancy compared to those with less severe disease [17]. Patients who have experienced the negative health effects of COVID-19 infection were willing to be vaccinated because they did not want to experience these symptoms again and would have a greater fear of reinfection. Previous studies also suggested that the severity perception of COVID-19 infection would directly affect the intention to vaccinate against COVID-19; patients who perceived COVID-19 infection to be severe were the most likely to be vaccinated [52,53]. In addition, patients with symptoms may develop sequelae symptoms and thus believe that they are vulnerable and that their perceived risk of COVID-19 infection will be higher, leading to the belief that they need to be vaccinated. Results of a cohort study showed that 61.4% of patients infected with the original SARS-CoV-2 strain had at least one sequelae symptom [54]. The significance of vaccination was not limited to preventing COVID-19 infection, as vaccination in COVID-19–recovered patients was found to also be effective at preventing sequelae symptoms [55]. Several studies have shown that the vaccinated group had a lower risk of developing sequelae symptoms compared to the unvaccinated group [56-58]. Emphasizing the benefits of vaccination among hesitant patients, such as prevention of reinfection and prevention of sequelae, will raise their awareness of the need for vaccination and thus motivate unvaccinated COVID-19–recovered patients to get vaccinated.

Complacency occurs when individuals have a lower perception of the need for a vaccination or a perceived low risk from diseases, which is influenced by general health beliefs [59,60]. Similar to other studies, complacency had a significant effect on increasing COVID-19 vaccine hesitancy in this study [61,62]. The immunity from previous infections might be one of the reasons for the complacency among the patients who recovered from COVID-19 infection. Meanwhile, misconceptions about the efficacy or safety of vaccination, misinformation in popular social media, and the perception that vaccines may not offer better protection than previous infections also could lead to complacency and result in underacceptance of the COVID-19 vaccine [63-65]. Additionally, the belief that vaccination of those around them is sufficient to prevent transmission and protect themselves from COVID-19 reinfection could result in a lower perception of COVID-19 reinfection risk among COVID-19–recovered patients. According to our study, among the reasons why COVID-19 survivors were willing to be vaccinated, most participants chose “self-needs,” which could eventually lead to high vaccine uptake [66]. By reducing their beliefs about complacency, this group of COVID-19 survivors might realize that vaccination is necessary for them. Specifically, health systems and relevant authorities should provide valuable information highlighting the evidence that immunity gained from natural infection would diminish over time and the risks of failure to vaccinate to enhance their “self-needs.” This would increase vaccination rates among COVID-19 survivors and ensure that they have sufficient protection against reinfection.

Convenience was a significant factor affecting vaccine hesitancy. Previous vaccination programs have shown that obstacles to vaccination include limited access to information, difficulties obtaining vaccines, unaffordable vaccine prices, and the long time (or distance) required to receive a vaccine [67-69]. Our results showed that “long waiting time for vaccination” and “have had a bad experience with the health clinic or provider” were among the convenience-related reasons participants stated for not receiving the COVID-19 vaccine. “Free vaccination” was among the reasons why participants received a COVID-19 vaccine, which is related to high convenience. Hence, to enhance COVID-19–recovered patients’ convenience in getting vaccinated for COVID-19, relevant health facilities should optimize the vaccination process to shorten waiting or queueing times, further improve the vaccination environment, and train vaccination-related workers to make them knowledgeable about the vaccine and to be more patient when answering questions [65]. In addition, targeting COVID-19–recovered patients with mobility issues and offering door-to-door vaccinations to address accessibility barriers would be very effective measures to increase vaccination rates.

Trust is a strong driver in reducing vaccine hesitancy. People with a higher level of trust in health authorities would have a more positive perception of the COVID-19 vaccine [70,71]. This study utilized two questions to evaluate participants’ trust, and the results also showed that trust was a motivating factor for patients who recovered from COVID-19 to get immunized. However, a crisis of trust in the COVID-19 vaccine, vaccine manufacturers, and health facilities unavoidably arose as a result of vaccine safety–related events and the dissemination of false information since the COVID-19 outbreak. Trust would build when people feel that health authorities possess knowledge and expertise; that the authorities take into account all relevant opinions; and that the authorities are transparent, honest, and open [72]. To assist in fostering trust and boosting confidence in the COVID-19 vaccine, identifying the concerns of patients who recovered from COVID-19, providing accurate information, and establishing communication channels with the health authorities are necessary. Ensuring appropriate, effective, and more specific education targeted at patients who recovered from COVID-19, while expressing the overall benefits and risks of the vaccine would also be beneficial to build trust to promote vaccination.

Since the availability of COVID-19 vaccines, we have dependable, high-quality evidence attesting to their safety, effectiveness, and value of protecting people from COVID-19 infection. However, our research has shown that patients are likely to be hesitant to receive vaccines because of complacency, convenience, trust, and other issues. In December 2022, a large-scale outbreak of COVID-19 infection occurred in China that lasted for 2-3 months, resulting in a high proportion of the population being in the category of COVID-19–recovered patients. These individuals will inevitably have to decide whether to get another dose (booster shot) if the COVID-19 epidemic persists and new variants continue to emerge. The study team conducted an online survey between January 5, 2023, and February 9, 2023, among people in seven geographic subdivisions of China regarding their willingness to receive a booster shot. A total of 7070 valid questionnaires were collected among these COVID-19–recovered patients and the COVID-19 vaccine hesitancy rate was 43.54% (data not shown). This high rate indicates that vaccine hesitancy still exists among COVID-19–recovered patients in China since the latest surge of the COVID-19 pandemic. The findings of this study may contribute to providing a research base and support for interventions to reduce future hesitancy related to COVID-19 booster shots among COVID-19–recovered patients, since future booster shots and the COVID-19 vaccination services and procedures that are currently in progress may converge.

### Limitations

This study has several limitations. First, the study was conducted in Wuhan, China, and a convenience sampling approach was employed for the survey, which may result in potentially biased estimates and selection bias. Therefore, the generalizability of our results will still be limited in certain aspects. Second, this was a cross-sectional study using a self-report questionnaire, leading to the presence of information bias. Third, since these data were collected between June and July 2021, the investigation was carried out in a very dynamic and ever-changing context, and it is possible that current perceptions of vaccines have changed both in terms of the perceived disease threat to study participants and the development of the COVID-19 vaccine itself.

### Conclusions

COVID-19–recovered patients may have a low perception of the risk of reinfection because they already have immunity acquired through natural infection, and this may lead to complacency, ultimately leading to COVID-19 vaccine hesitancy. Advocacy strategies based on scientific evidence to raise public awareness of the risk of reinfection and the superiority of immunity gained from vaccination over that gained from infection can be effective in reducing complacency and thus overcoming vaccine hesitancy. Improving convenience-related factors such as time, transportation, and environment to get vaccinated is also necessary to address accessibility barriers and facilitate vaccination uptake. Furthermore, education efforts targeted at individuals who recovered from COVID-19 based on solid and adequate knowledge are needed to address their concerns about vaccination, which could foster greater trust and promote their vaccination acceptance.

## Abbreviations

AVE: average variance extracted
CFI: comparative fit index
CR: composite reliability
OR: odds ratio
REDCap: Research Electronic Data Capture
RMSEA: root mean square error of approximation
TLI: Tucker Lewis index
WHO: World Health Organization
3Cs: complacency, convenience, confidence

## Data Availability

The data sets generated during and/or analyzed during the current study are available from the corresponding author on reasonable request.
